# Absence of Diabetes and Pancreatic Exocrine Dysfunction in a Transgenic Model of *Carboxyl-Ester Lipase*-MODY (Maturity-Onset Diabetes of the Young)

**DOI:** 10.1371/journal.pone.0060229

**Published:** 2013-04-02

**Authors:** Helge Ræder, Mette Vesterhus, Abdelfattah El Ouaamari, Joao A. Paulo, Fiona E. McAllister, Chong Wee Liew, Jiang Hu, Dan Kawamori, Anders Molven, Steven P. Gygi, Pål R. Njølstad, C. Ronald Kahn, Rohit N. Kulkarni

**Affiliations:** 1 Joslin Diabetes Center, Harvard Medical School, Boston, Massachusetts, United States of America; 2 KG Jebsen Center for Diabetes Research, Department of Clinical Medicine, University of Bergen, Bergen, Norway; 3 Department of Pediatrics, Haukeland University Hospital, Bergen, Norway; 4 Center for Medical Genetics and Molecular Medicine, Haukeland University Hospital, Bergen, Norway; 5 Department of Cell Biology, Harvard Medical School, Boston, Massachusetts, United States of America; 6 The Gade Institute, University of Bergen, Bergen, Norway; 7 Department of Pathology, Haukeland University Hospital, Bergen, Norway; 8 Broad Institute of Harvard and MIT, Cambridge, Massachusetts, United States of America; University of Texas Health Science Center at San Antonio, United States of America

## Abstract

**Background:**

CEL-MODY is a monogenic form of diabetes with exocrine pancreatic insufficiency caused by mutations in *CARBOXYL-ESTER LIPASE* (*CEL*). The pathogenic processes underlying CEL-MODY are poorly understood, and the global knockout mouse model of the CEL gene (CELKO) did not recapitulate the disease. We therefore aimed to create and phenotype a mouse model specifically over-expressing mutated *CEL* in the pancreas.

**Methods:**

We established a monotransgenic floxed (flanking LOX sequences) mouse line carrying the human *CEL* mutation c.1686delT and crossed it with an elastase-Cre mouse to derive a bitransgenic mouse line with pancreas-specific over-expression of *CEL* carrying this disease-associated mutation (TgCEL). Following confirmation of murine pancreatic expression of the human transgene by real-time quantitative PCR, we phenotyped the mouse model fed a normal chow and compared it with mice fed a 60% high fat diet (HFD) as well as the effects of short-term and long-term cerulein exposure.

**Results:**

Pancreatic exocrine function was normal in TgCEL mice on normal chow as assessed by serum lipid and lipid-soluble vitamin levels, fecal elastase and fecal fat absorption, and the normoglycemic mice exhibited normal pancreatic morphology. On 60% HFD, the mice gained weight to the same extent as controls, had normal pancreatic exocrine function and comparable glucose tolerance even after resuming normal diet and follow up up to 22 months of age. The cerulein-exposed TgCEL mice gained weight and remained glucose tolerant, and there were no detectable mutation-specific differences in serum amylase, islet hormones or the extent of pancreatic tissue inflammation.

**Conclusions:**

In this murine model of human *CEL*-MODY diabetes, we did not detect mutation-specific endocrine or exocrine pancreatic phenotypes, in response to altered diets or exposure to cerulein.

## Introduction

Mutations in the *CARBOXYL-ESTER LIPASE* (*CEL*) gene cause a monogenic syndrome of diabetes (Maturity onset diabetes of the young type 8; OMIM #609812) and pancreatic exocrine dysfunction [1] with fat infiltration of the pancreases as reported in mutation-carrying subjects with [1] or without [2] diabetes. The pathophysiological mechanisms leading to the disease phenotype are evasive, although protein misfolding may play a role in the pathogenic process [3]. We have previously reported that a global *Cel* knockout mouse model (CELKO) [4], [5] did not display features of pancreatic exocrine dysfunction or diabetes [6], suggesting a negative gain-of-function effect rather than a simple loss-of-function of CEL enzyme activity. This is supported by functional and cellular data [3]. In the present work, we used the cre-lox system [7] and the elastase-cre mouse line [8] to engineer TgCEL mice selectively expressing a human *CEL* mutation (1686delT) [1] in the pancreas to study the potential pathophysiological effects. Furthermore, we aimed to phenotype these mice when fed normal chow and compared with a high fat diet challenge [6]. In a second series of experiments, we explored the consequences of exposure of the TgCEL model to cerulein, as the latter has been successfully used to elicit murine pancreatic phenotypes in other disease models of monogenic pancreatic disease [9]. Surprisingly, in the TgCEL model, we did not detect differences in glucose tolerance, pancreatic exocrine function or pancreatic morphology in response to a HFD or cerulein challenge suggesting that other approaches are necessary to unmask a phenotype.

## Methods

### Ethics Statement

All protocols (Protocol number: 05-01, Protocol Title: Phenotyping mouse models of diabetes and insulin resistance) for animal use and euthanasia were approved by the Animal Care Use Committee of the Joslin Diabetes Center and Harvard Medical School in accordance with National Institutes of Health guidelines.

### Constructs

**Figure 1:**
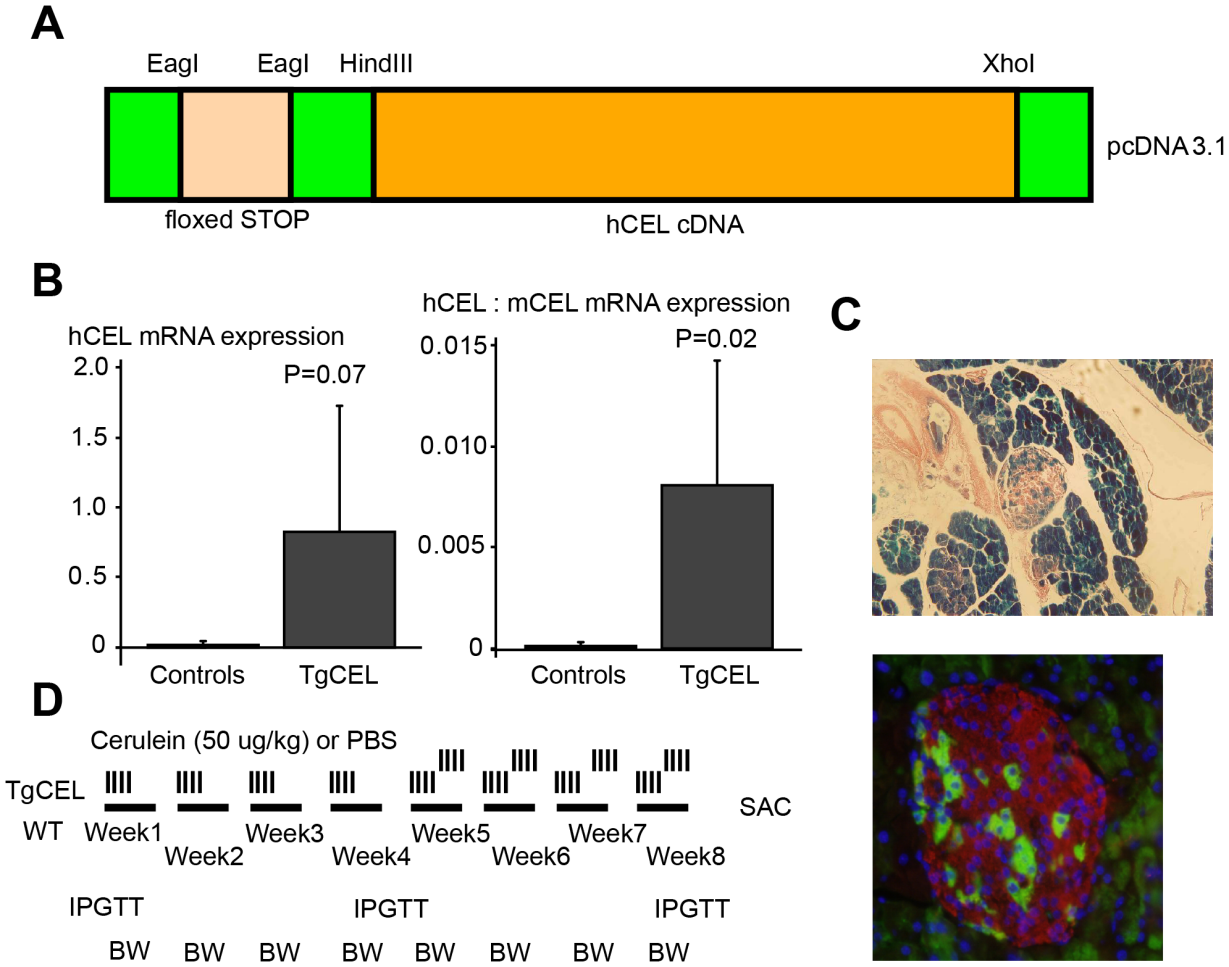
Engineering of a TgCEL mouse model and the chronic cerulein protocol. (A) Structure of the construct that was linearized and subjected to pronuclear injection into fertilized C57BL/6 mice. (B) Pancreatic mRNA expression of human carboxyl-ester lipase, h*CEL,* normalized to beta actin in TgCEL mice compared to controls (left panel) and the relative expression of *hCEL* to murine *Cel* (mCEL) in TgCEL mice compared to controls (right panel). (C) X-gal staining (blue; upper panel) and immunofluorescent histochemical analysis (lower panel) of pancreas sections of bitransgenic *LacZ*+/−,elastase-Cre+/− mice confirmed induction by the elastase promoter of *Cre* expression in acinar tissue and in 10–15% of β cells in pancreatic islets from two different mice (Green, *LacZ*; red, insulin; blue, nuclei). (D) Chronic cerulein protocol; PBS, Phosphate buffered saline; WT wild type; IPGTT, intraperitoneal glucose tolerance test; BW, body weight.

We excised the wild-type *CEL* sequence (cDNA) from a pBS-vector using the restriction enzymes *Hin*dIII and *Xho*I and cloned into the *Hin*dIII/*Xho*I site of the expression vector pcDNA3.1 (Invitrogen, City, CA). See Figure 1A for the restriction enzyme sites. By mutagenesis an *Nhe*I restriction enzyme site was converted to an *Not*I site and furthermore to an *Eag*I site. Using the restriction enzyme *Eag*I we cloned a floxed STOP sequence from a pBS302 vector into this site. Cloning of the disease haplotype exon 11 sequence of an affected individual (III-9 of Family 1 in ref. [1]) into the consensus cDNA sequence of the expression vector was performed to create the mono-transgenic floxed STOP-CEL mutant mouse line. By *in vitro* mutagenesis (QuickChange XL kit, Stratagene, La Jolla, CA) we introduced into the pcDNA3.1-CEL construct a C->T mutation at position c.1513, creating a novel restriction site for *Bam*H1 in the N-terminal part of exon 11 without affecting the amino acid sequence, using the primers 5′-ccaaaacaggggatcccaacatgggcgac-3′ and 5′-gtcgcccatgttgggatcccctgttttgg-3′. Primers were designed to introduce the corresponding *Bam*HI/*Xho*I restriction sites at position c.1513/+117 in the PCR template from amplification of exon 11 of the family member III-9 (5′-GCAGGGATCCCAACATGG-3′, 5′-tactcGAGCAAAGAAAGACACCGACAG-3′). The template was digested with *BamHI*/*Xho*I and ligated into the restriction sites in exon 11, introducing the exact sequence of the affected family members, harboring a sequence with 14 variable number of tandem repeats in the C-terminal part instead of the 16 consensus repeats and in addition the mutation, c.1686delT.

### Creation of Transgenic Mice

A transgenic floxed STOP-CEL mouse line was created at the Beth Israel Transgenic Mouse Facility (Boston, MA) by standard transgenic protocols using pronuclear injection of the construct (see above) into fertilized C57BL/6 mice. The elastase-Cre mouse line on C57BL/6 background was provided by Dr. Grippo [8]. The monotransgenic floxed STOP-Rosa26 mouse line [10] on C57BL/6 background was purchased from Jackson Laboratories (Bar Harbor, ME). All animals were housed on a 12 hr light-dark cycle and fed a standard rodent chow.

### Genotyping of Mice by PCR

Genotype analysis was done by PCR analysis of tail-tip-derived DNA samples. For the elastase-Cre mouse line, the following primers were synthesized to differentiate wild-type mice from mice carrying EL-Cre: Cre F 5′ ccgtttgccggtcgtggg 3′ and Cre R 5′ cgtatatcctggcagcga 3′ to amplify a band of 337 bp in mice carrying EL-Cre. For the floxed STOP-CEL mouse line, the primers CEL-HIS-2F: 5′ gcctgcaactggttgtgt t 3′ and CEL-HIS-2R: 5′ gtggacatcttcaagggc at 3′ were used to amplify a band of 141 bp in mice carrying the construct. For the Rosa26 mouse lines, the primers R26F: 5′ ggcttaaaggctaacctgatgtg 3′, R26LR: 5′ gcgaagagtttgtcctcaaac 3′ and R26R: 5′ ggagcgggagaaatggatatg 3′ amplified a fragment of 374 bp in WT mice and a fragment of 1146 in mice carrying the Rosa26 construct. For the *Cel* genotype analysis, each PCR reaction (25 µL) contained 1 µL of genomic DNA, 600 nM each primer, 400 µM of each deoxynucleotide triphosphate,1 M betain, 1 x GC-buffer and 1 unit of Lataq polymerase (Takara Shuzo, Otsu, Japan). The reaction mixture was heated to 95°C for 7 min and then subjected to 35 cycles of amplification consisting of 30 s at 95°C, 30 s at 58°C, and 30 s at 72°C. Samples were analyzed on a 2% agarose gel.

### Verification of Specificity of Elastase Promoter in Rosa26 Mice

Monotransgenic floxed STOP-Rosa26 mice on C57BL/6 background were crossed with elastase-Cre mice to create bitransgenic offspring that were genotyped and subsequently dissected. Thin tissue slices from pancreas, liver, spleen, epididymal fat, small intestines, colon, hypothalamus and testes/ovaries were fixed for 4 hours in formalin, stained overnight in x-gal and then transferred to 70% ethanol. Fixed tissue was paraffin-embedded and eosin-stained sections were used for direct microscopic examination. Sections of pancreas were also prepared by immunohistochemical staining with antibodies against *LacZ* (#55976, MP Biomedicals, Irvine, CA; and biotinylated donkey anti-rabbit, Jackson Labs, Bar Harbour, ME) and insulin (# 4011-01, Linco Research, St Charles, MO; and donkey anti-guinuea pig-Texas red secondary antibody, Jackson Labs).

### Assessment of *CEL* Gene Expression in TgCEL Mice by Quantitative PCR

Total RNA was extracted using an RNeasy mini kit (Qiagen), and RNA was reverse transcribed using a high capacity cDNA reverse transcription kit (Applied Biosystems, Foster City, CA) according to the manufacturer’s instructions. *Cel* gene expression was determined by quantitative Real-Time PCR [11]. Primer information is provided in Supporting Information S1. Fluorescence was monitored and analyzed in an ABI Prism 7000 sequence detection system (Applied Biosystems). Analysis of beta-actin expression was performed in parallel to normalize gene expression.

### High Fat Challenge

To produce insulin resistance that might challenge the TgCEL mice, mice were placed on a high fat diet consisting of 60% fat, 20% carbohydrate and 20% protein (% of kcal) (“60% HFD”; males [n = 4 TgCEL+/−,Cre+/− and 4 controls] and females [n = 5 TgCEL+/−,Cre+/− and 5 controls], 9 months old at baseline). The diet was provided for 12 weeks prior to physiological testing (the 12 months age group). After this, the HFD was terminated and the mice reverted to normal chow until testing was repeated at 22 months of age in the remaining animals (males: n = 1 TgCEL+/−,Cre+/− and 2 controls, females: n = 5 TgCEL+/−,Cre+/− and 3 controls).

### Cerulein Challenge

Acute pancreatitis was induced in TgCEL mice and controls of either sex by 6 hourly intraperitoneal injections of 50 µg/kg cerulein (Sigma, St Louis, MO) [12] (short-term exposure). Chronic pancreatic injury was induced in only female TgCEL mice and controls by one series of four injections of 50 µg/kg cerulein per week for four weeks, subsequently followed by two series of four injections of 50 µg/kg cerulein per week for four weeks (Fig 1D) (long-term exposure). Control mice were injected with the same volume of phosphate buffered saline (PBS).

### Test Groups

The group of mice tested under basal conditions at seven months of age (males [n = 4 TgCEL+/−,Cre+/− and 4 controls] and females [n = 5 TgCEL+/−,Cre+/− and 5 controls]) were investigated at Joslin Diabetes Center, Boston, USA. Another group of mice were tested with high fat diet challenge in Bergen, Norway, at several time points: Nine months of age (baseline; males [n = 4] and females [n = 5]), after a 12 weeks high fat challenge at 12 months of age (males [n = 4] and females [n = 5] ), and at 22 months of age in the remaining animals (males [n = 1 TgCEL+/−,Cre+/− and 2 controls], females [n = 5 TgCEL+/−,Cre+/− and 3 controls]). A third group of mice were used only for islet isolation.

The remaining two groups of mice tested with cerulein challenge were tested with acute exposure at the age of 12–17 months (n = 6 TgCEL+/−,Cre+/− mice exposed to cerulein; males [n = 3] and females [n = 3], n = 6 TgCEL+/−,Cre+/− mice exposed to PBS; males [n = 3] and females [n = 3], n = 6 control mice exposed to cerulein; males [n = 2] and females [n = 4], n = 6 control mice exposed to PBS; males [n = 2] and females [n = 4]) or chronic exposure at 4–6 months of age [only female mice; n = 8 TgCEL+/−,Cre+/− mice exposed to cerulein, n = 8 TgCEL+/−,Cre+/− mice exposed to PBS, n = 8 control mice exposed to cerulein, n = 8 control mice exposed to PBS) as explained above. These groups of mice were investigated at Joslin Diabetes Center, Boston, USA.

### Physiological Tests

We measured fed and fasting blood glucose levels by a Glucometer Elite (Bayer Health Care) using blood from tail snips. For other analyses, blood was collected in chilled heparinized tubes and centrifuged (5–10 min at 5000 rpm) and the supernatants were collected and stored at −20°C. Plasma insulin and glucagon levels were measured by ELISA using mouse insulin and glucagon standards, respectively (Crystal Chem Inc., Chicago, IL). Blood glucose and plasma insulin levels were measured in the random-fed state between 8∶30 and 11 am or after a 14–16-hr overnight fast. Serum triglyceride levels were measured by colorimetric enzyme assay (GPO-Trinder Assay; Sigma, St. Louis, MO). Free fatty acid levels were measured using the NEFA-Kit-U (Amano Enzyme, Osaka, Japan). Serum amylase was measured by an enzyme assay (Raichem, San Diego, CA).

For glucose tolerance tests (intraperitoneal glucose tolerance tests, ipGTT), mice were fasted overnight (14 hr) and then received intraperitoneal (i.p.) injections of glucose (2 g/kg body weight [b.wt.]). Tail vein glucose was measured as described above at 0, 15, 30, 60, and 120 min after injection. For insulin tolerance tests (ITT), fed mice received i.p. injections of insulin (1 U/kg b.wt. for females, 1.5 U/kg b.wt. for males; Humulin, Lilly, Indianapolis, IN) and tail vein glucose was measured at 0, 15, 30, and 60 min after injection. Stimulated acute-phase insulin secretion tests were performed after an overnight fast (14 hr). Tail vein glucose was measured and blood for insulin analysis collected and treated as described above at 0, 15, 30 and 60 min after an ip injection of either glucose (3 g/kg b.wt.; GSIS) or a combination of glucose in the same dose as above and arginine (0.3 g/kg b.wt., Arg-GSIS).

Fat malabsorption was measured by comparing the fecal excretion of fat and the non-absorbable dietary marker sucrose polybehenate to the ratio of behenic acid to other dietary fatty acids, using gas chromatography analysis as previously described [13]. In addition, the percent fat excretion in stool after a four-day high fat challenge, was measured. The enzymatic activity of elastase in mouse feces was analyzed by fluorometry as described in [14].

### Islet Isolation and Assessment of Gene Expression

Islets were obtained by collagenase digestion as previously described [15] and were maintained in RPMI-1640 media supplemented with 7 mM glucose and 10% v/v FCS. Gene expression studies were performed in healthy handpicked islets. After overnight incubation, total RNA was extracted using RNeasy Mini Kit (Qiagen). Total RNA was also extracted from pancreata that were snap frozen in liquid nitrogen at dissection. cDNA were generated by reverse transcriptional DNA synthesis (Applied Biosystems, Foster City, CA). Gene expression for murine *Cel*, glucagon, insulin, Glut2, Pdx-1, Mafa, Neuro-D1 and amylase was determined by quantitative Real-Time PCR [11] using appropriate primers (Supplementary Methods), and normalized for beta-actin.

### Morphology and Tissue Preparation

Weight and glucose levels were decided in fed mice before they were anesthetized and ex-sanguinated. Blood was collected and prepared as described above. The pancreas was rapidly dissected and divided into two or three parts. Pancreatic samples to be studied by light microscopy were weighed, fixed in pre-chilled zinc-formalin (Z-Fix), then paraffin-embedded, sectioned, hematoxylin and eosin (HE)-stained, and examined by direct microscopy. Other sections were prepared by immunohistochemical staining for amylase or for insulin, glucagon and somatostatin. Tissue samples for RNA extraction were either directly homogenized in tissue lysis buffer (pancreas), put in RNAlater (Ambion) (liver) or snap frozen (other tissues) and then stored briefly at −80°C.

### Immunohistochemistry

Immunohistochemical analyses of pancreas sections were performed by methods previously described [16]. Antibodies to the following proteins were used: Amylase (# sc-12821,Santa Cruz; and donkey anti-goat cy2 conjugated secondary antibody, Jackson Labs), insulin (# 4011-01, Linco Research,; and donkey anti- guinea pig-Texas red secondary antibody, Jackson Labs), glucagon (# G2654, Sigma; and donkey anti-mouse-Texas red secondary antibody, Jackson Labs), somatostatin (# n1551, DAKO; and donkey anti-rabbit cy2 conjugated secondary antibody, Jackson Labs).

### Statistical Analysis

We employed two-tailed Student’s t-tests of independent groups with assumption of unequal variances and a significance level of 5%. For the glucose tolerance tests, we performed analyses of variance (ANOVA) for repeated measures, using baseline measurements as a co-variate. For the comparison of the characteristics of the groups of mice after cerulein exposure, we also performed one-way analysis of variance (ANOVA) and used Bonferroni correction for post hoc analyses. Due to non-normal distribution, we used the Mann-Whithney U test for comparisons of islet area. We chose a significance level of 5% and analyzed all data using Stata 11.0 (Stata Statistical Software, Stata Corp., College Station, TX, USA).

## Results

### Engineering of a TgCEL Mouse Model

We used the Cre-lox system [7], [17] to create a murine pancreas-specific *Cel* over-expressing model, the TgCEL, for CEL*-*MODY by mating floxed STOP-CEL mice to elastase-Cre mice as described in Methods and Fig. 1A. TgCEL (TgCEL+/−,Cre+/−) mice of both sexes were compared to sex-matched littermate control mice of either of the following genotypes: TgCEL+/−,Cre−/− TgCEL−/−,Cre+/− TgCEL−/−,Cre−/−. There were no differences in body weights, fasting or random fed blood glucose values, serum insulin or glucose tolerance between the three control genotypes (data not shown). The correct genotypes of the bitransgenic animals were verified by PCR, and over-expression of the human *CEL* transgene in TgCEL mice was confirmed by quantitative PCR (Fig. 1B). Cross-mating of elastase-Cre mice with mice from a monotransgenic floxed STOP-Rosa26 mouse line harboring dormant *LacZ* gene confirmed the expression of Cre limited to the pancreas, most abundantly the pancreatic exocrine cells, but also ∼10% of the beta-cells (Fig. 1C). Overall, only 11% of the offspring of monotransgenic floxed STOP-CEL mice x elastase-Cre matings had the TgCEL+/−,Cre+/− (TgCEL) genotype, while 25% would be expected from Mendelian laws, suggesting some lethality *in utero*. The time points for the characterizations of the TgCEL mice under normal chow and following 60% HFD and cerulein exposure are outlined in Methods and in Figure 1D.

### Characterization of the TgCEL Mice under Basal Conditions

**Figure 2:**
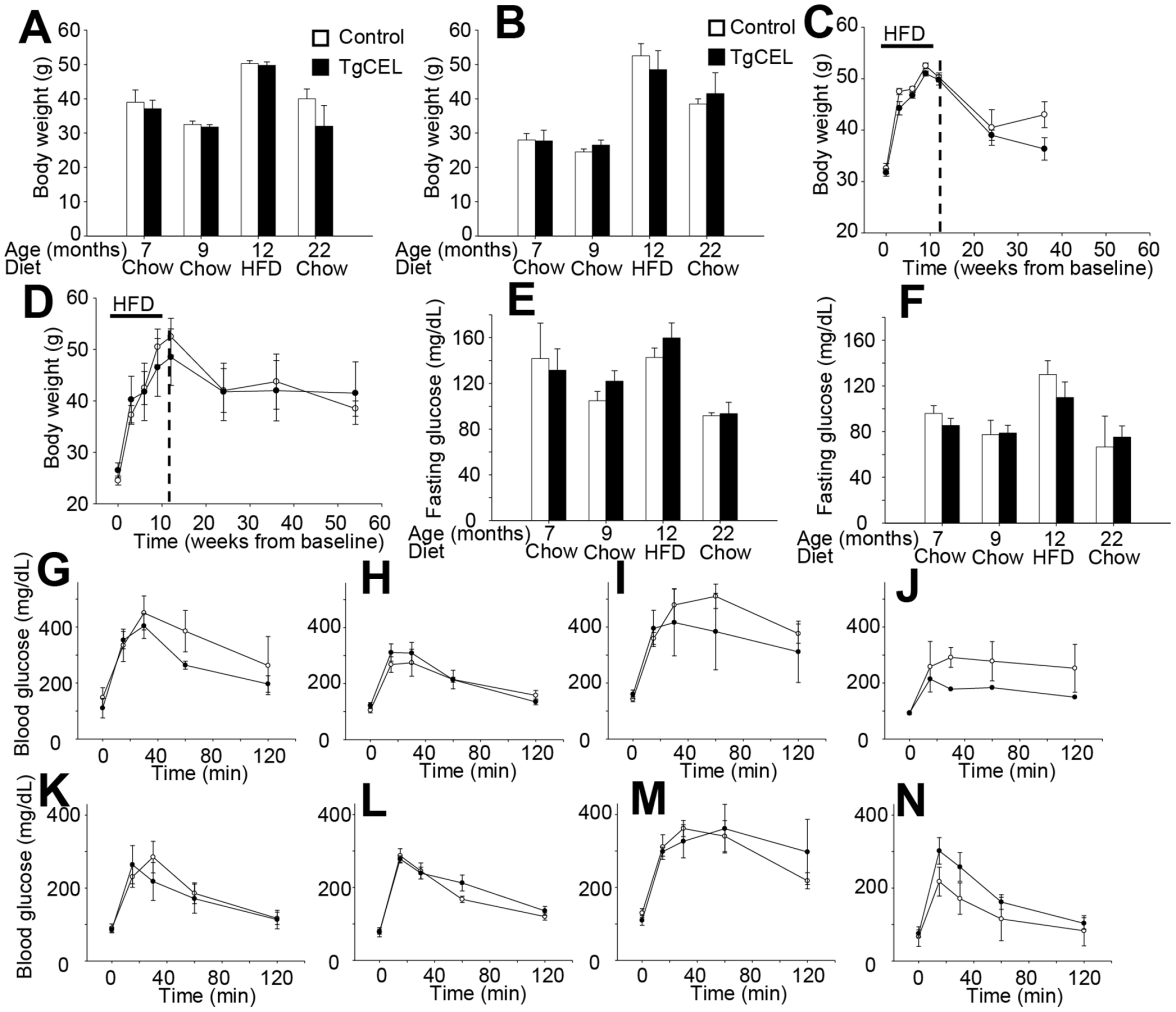
Weight and glucose homeostasis in TgCEL and control mice. Controls, white bars or open circles; TgCEL mice, black bars or filled circles. Results are given as mean ± SEM. Tail or hind leg vein blood was drawn in after an overnight fast and glucose was measured by glucometer. The characteristics were assessed in chow-fed mice at 7 and 9 months of age, at 12 months of age following 12 weeks on a 60% HFD (all tests: n = 4 [males] and n = 5 [females]), and at 22 months of age in mice that had been chow-fed after the HFD challenge (n = 2 controls, 1 TgCEL [males] and n = 3 controls, 5 TgCEL [females]). There were no statistical differences in body weight between male (A) and female (B) mice. Body weights increased during a 12-week challenge with a 60% HFD, but there were no differences in body weight development between TgCEL and control mice in male (C) and female mice (D). Fasting blood glucose levels also increased after 12 weeks of HFD but there were no differences in fasting blood glucose levels between TgCEL and control mice at any age in male (E) and female mice (F). A glucose tolerance test was performed in male mice (G, H, I, J) and female mice (K, L, M, N) after a 12–14 hr fast by i.p. injection of glucose (2 g per kg bodyweight). The test was performed in chow-fed mice at 7 months (G, K) and 9 months (H, L) of age, at 12 months (I, M) of age following 12 weeks of a 60% HFD. Although the 60% HFD increased glucose intolerance compared to baseline (9 months), there were no differences between TgCEL mice and controls in any of the groups.

The TgCEL and control mice showed comparable body weights during development, and a relatively normal life span and behaviour. At seven and nine months of age, there were no significant differences in mean body weights between TgCEL and control mice (Fig. 2A,B). Exocrine pancreatic function was normal in mice fed normal chow as assessed by serum lipid and lipid-soluble vitamin levels, serum amylase levels and fecal elastase levels (Table 1). No differences in fecal fat absorption were observed as measured by the ratio of excreted lipids in the stool to excreted sucrose behenate, a dietary marker. Furthermore, mass spectrometry-based proteomics methods revealed no significant differences in protein expression in pancreatic lysates of TgCEL and control mice (Supporting Information S2).

**Table 1:** Pancreatic exocrine function in chow-fed TgCEL and control mice at 7 months of age.

	Males			Females		
	Controls	TgCEL	*P*	Controls	TgCEL	*P*
*Serum lipid levels*						
Total serum cholesterol (mg/dL)						
Fasting	125±17	106±5	ns	88±5	92±10	ns
Fed	163±13	158±31	ns	122±26	135±21	ns
Serum triglycerides (mg/dL)						
Fasting	107±13	92±8	ns	77±14	74±12	ns
Fed	119±18	111±16	ns	70±23	71±22	ns
Free fatty acids (mEq/L)						
Fasting	1.6±0.1	1.4±0.2	ns	1.0±0.1	NA	
Fed	1.3±0.2	1.1±0.2	ns	1.0±0.2	0.9±0.2	ns
*Exocrine pancreatic function*						
Serum amylase, fasting	1344±114	1197±101	ns	1057±90	NA	
Fat absorption (%)	94±2	93±2	ns	98±0,46	99±0,27	ns
Fat excretion (%) [60%HFD, n = 2]	4.5±1.0	8.1±0.9	ns	7.5±1.6	6.0±0.1	ns
Fecal elastase (milliU/g)	8.9±3.0	7.5±2.1	ns	14.2±4.7	21.1±7.4	ns
Vitamin A, retinol (µg/dL)	38±7	30±3	ns	18±1,2	18±0,42	ns
Vitamin E, α-tocopherol (µg/dL)	387±37	371±22	ns	462±30	432±32	ns
Vitamin E/total cholesterol ratio	3.0±0.4	2.6±0.3	ns	3.6±0.23	3.7±0.72	ns

Values are mean ± SEM; ns, not significant; NA, not available.

### TgCEL Mice have Normal Pancreatic Exocrine Function and Remain Glucose Tolerant on a High Fat Diet

**Figure 3:**
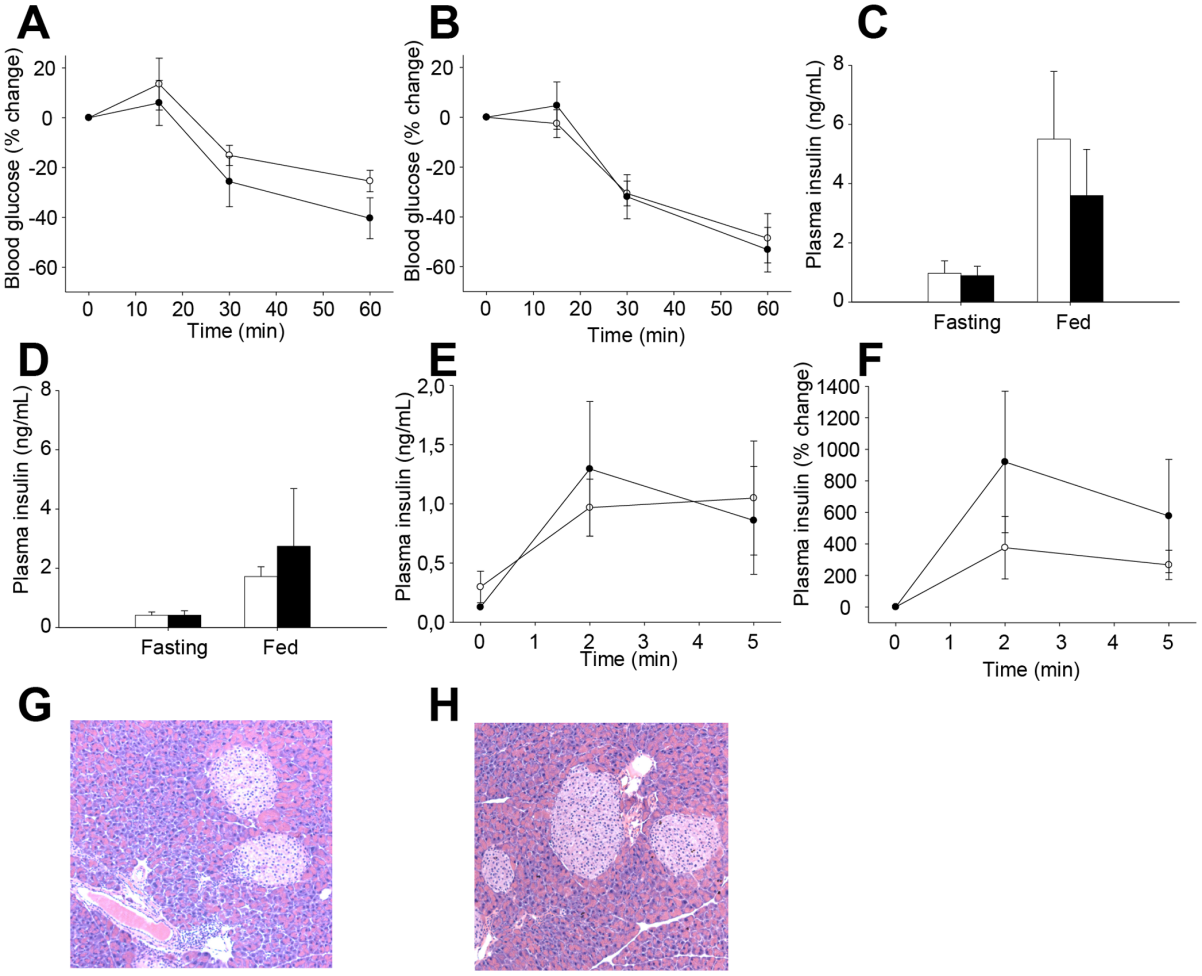
Insulin homeostasis and pancreatic morphology in TgCEL and control mice. Controls, white bars or open circles; TgCEL mice, black bars or filled circles. Results are given as mean ± SEM. Insulin was measured by ELISA. An insulin tolerance test was performed by i.p. injection of insulin (1.5 [males] or 1.0 [females] U/kg b.wt.) in 12 months old male (A) and female (B) mice. No differences were detected between the TgCEL and control mice. For 7 months old chow-fed mice, there were no statistical differences in fed or fasting plasma insulin levels in male (C) or (D) female mice. Similarly, we observed no differences in stimulated plasma insulin levels in female mice as demonstrated by a glucose-stimulated insulin secretion test by actual insulin values (E) or by percent change after glucose injection (F). Islet size and number as well as the organization of the exocrine pancreatic tissue were normal in HE-stained sections from TgCEL mice (G) and not different from controls (H) at 8 months of age.

Following a 60% HFD, TgCEL mice gained weight to the same extent as controls (with a weight gain in male mice of 57% for TgCEL and 55% for control mice, and in female mice of 81% in TgCEL and 114% in control mice; *P* = 0.09; Fig. 2A,B) and also on resuming normal diet until a follow up of 22 months of age (Fig. 2A,B). There were no significant differences in fecal elastase levels after the HFD (controls, 45.4±14; TgCEL, 90.6±25.3; *P* = 0.12). Furthermore, fecal fat excretion after four days of high fat feeding was not significantly different between TgCEL mice and controls (not shown). There were no significant differences in fasting blood glucose levels between TgCEL and control mice at any of the time points (Figure 3E,F). Random fed blood glucose did not differ between TgCEL and control mice on normal chow or after the HFD (data not shown). Although HFD induced glucose intolerance, there were no significant differences in tolerance between TgCEL and control groups at any of the time points (Figure 2G–N). Insulin sensitivity measured by an insulin tolerance test showed no significant difference between TgCEL mice and controls after 12 weeks on a 60% HFD (Fig 3A,B). There were no statistical differences in fed or fasting plasma insulin levels (Fig. 3C,D) or in fasting C-peptide levels (data not shown) or in the insulin secretory response as demonstrated by a glucose-stimulated insulin secretion test in seven months old chow-fed mice (Fig. 3E,F). Random fed glucagon levels showed no significant differences (data not shown). Pancreatic morphology was normal in HE-stained sections (Fig 3. G,H). Analysis of sections of pancreas using a cocktail of antibodies to islet hormones showed no apparent differences in the ratio of β- to non-β cells at eight months of age (data not shown). Assessments of acinar and islet cell morphology by electron microscopy revealed a well-preserved architecture of both cell types in both TgCEL and control mice (data not shown).

### No Changes in Pancreatic Function or Morphology of TgCEL Mice after Short-term Cerulein Exposure

**Figure 4:**
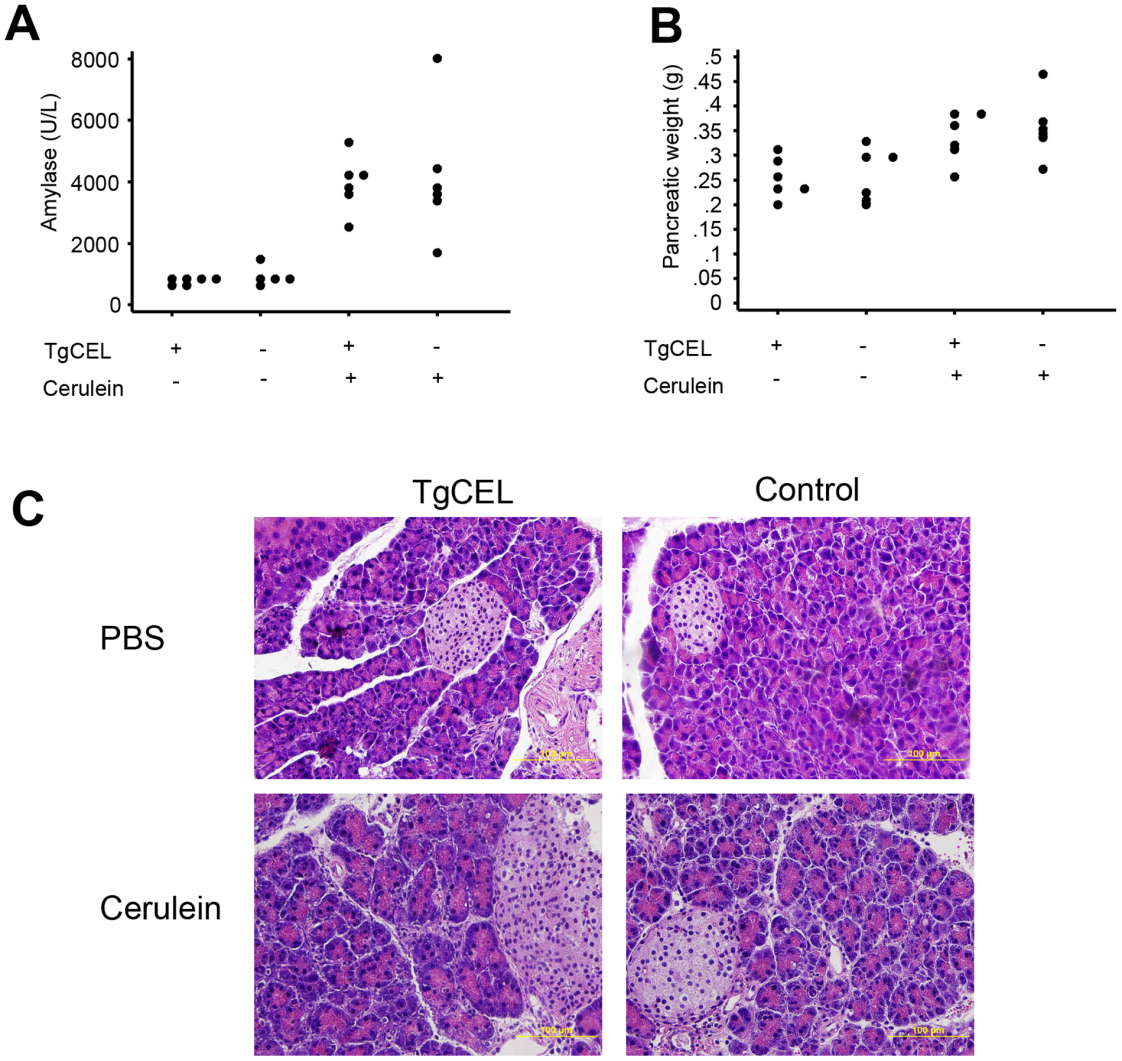
Characteristics of mice subjected to the short-term cerulein protocol. (A) Serum amylase levels by the time of sacrification of the mice. (B) Pancreatic weight (grams) by the time of sacrification of the mice. (C) Pancreatic morphology in TgCEL or Control mice injected with cerulein or with PBS (HE staining).

There were no significant differences in serum glucose levels prior to short-term cerulein exposure between the four groups TgCEL/PBS, Control/PBS, TgCEL/Cerulein and Control/PBS (ANOVA p-value 0.56, mean glucose levels of 71±12, 65±8.9, 65±18, 73±8.8 mg/dl for the four groups, respectively). The serum amylase levels and pancreatic weight increased in the mice exposed to cerulein (ANOVA p<0.05) but there were no significant differences between the TgCEL and control mice (Figure 4A,B; post hoc Bonferroni p-values >.05). There were also no significant differences in serum glucose levels after cerulein exposure between TgCEL and control mice (post hoc Bonferroni p-value>0.05; mean glucose levels of 87±15, 88±33, 62±19, 54±10 mg/dl for the four groups, respectively). Morphological analysis of pancreatic HE sections revealed no differences between the TgCEL and control mice (Figure 4C).

### No Changes in Pancreatic Function or Morphology of TgCEL mice After Long-term Cerulein Exposure

**Figure 5:**
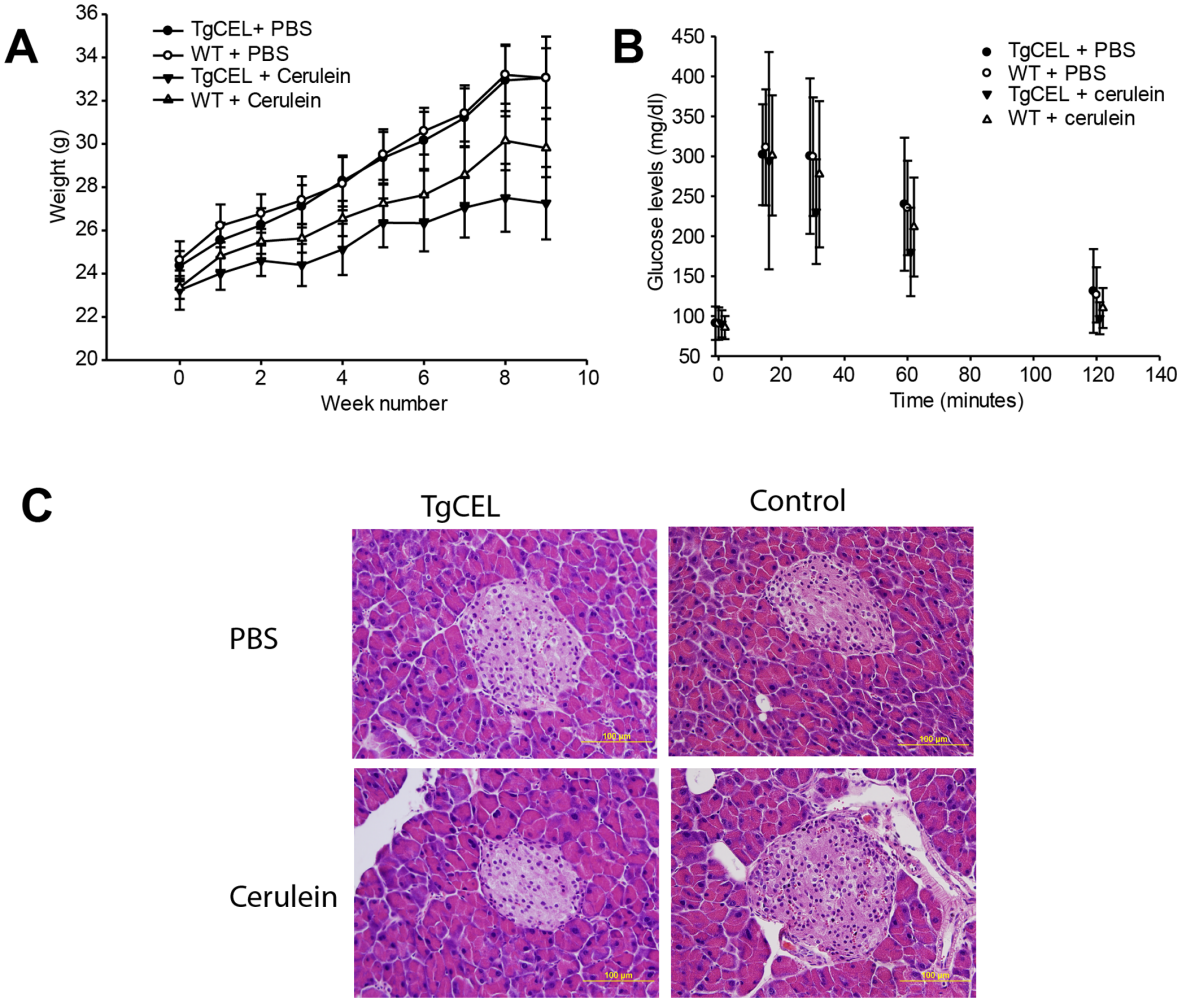
Characteristics of mice subjected to the long-term cerulein protocol. (A) Weight (grams) development during the chronic cerulein protocol in TgCEL mice and control mice subjected to cerulein or PBS. (B). Serum glucose levels during an intraperitoneal glucose tolerance test at the time of completion of the chronic cerulein protocol. (C) Pancreatic morphology in TgCEL or control mice injected with cerulein or with PBS (HE staining).

At the end of the long-term cerulein exposure there was a borderline significant difference in weight development between the four groups TgCEL/PBS, Control/PBS, TgCEL/Cerulein and Control/PBS, although post hoc analyses did not reveal significant differences between any of the groups (Figure 5A; ANOVA p-value 0.04, Bonferroni post-hoc p-values >.05; mean body weight of 33±5.4, 33±3.9, 27±4.8, 30±3.8 g for the four groups, respectively). There were no significant differences in serum glucose levels between the four groups during an intraperitoneal glucose tolerance test by the end of the cerulein exposure (Figure 5B, ANOVA p-value >.05), and no differences at baseline or after four weeks (data not shown). Serum levels of insulin, C-peptide, glucagon, GLP-1, amylin or leptin were not different between groups at the end of the cerulein exposure (Table 2). Morphological analysis of pancreatic HE sections revealed no differences between the TgCEL and control mice (Figure 5C).

**Table 2:** Serum levels of insulin, C-peptide, glucagon, GLP-1, amylin and leptin after long-term cerulein exposure.

	PBS		Cerulein		
	TgCEL	Control	TgCEL	Control	ANOVA p
*Serum levels*					
Insulin(pg/mL)	638±751	194±103	413±321	3668±9828	0.49
C-peptide (pM)	490±385	455±423	430±194	328±199	0.77
Glucagon(pg/mL)	46±26	36±11	46±30	75±62	0.21
GLP-1 (pg/mL)	7±10	20±28	7±11	19±30	0.51
Amylin(pg/mL)	14±7	13±9	18±6	16±9	0.63

### Islet Isolation and mRNA Expression

We isolated islets from eight TgCEL mice (four males, four females; 4–6 months of age) and sex-matched littermate controls under basal conditions and extracted RNA after an overnight incubation as described in *Methods*. We found no differences in expression levels for insulin, glucagon, glucokinase (the rate-limiting enzyme of glycolysis), the glucose transporter Glut2, or any of the transcription factors Pdx-1, Neuro-D1, MafA and Hnf-1α, which are all involved in beta cell differentiation and regulation (data not shown). As indicators of the fat metabolism pathway, we chose to investigate the expression of Srebp and Dgat1, but found no differences in their expression between groups (data not shown).

## Discussion

We have created an over-expressing transgenic mouse model, TgCEL, to study *CEL*-MODY, a human monogenic syndrome of diabetes and pancreatic exocrine dysfunction caused by mutations in the gene encoding carboxyl-ester lipase [1]. To mimic this disease we crossed mice with floxed mutated human *CEL* (1686delT) to elastase-Cre mice to produce TgCEL. First, however, we verified pancreatic specificity of Elastase-Cre mice by crossing these mice to Rosa26 mice [10] (with floxed lacZ to monitor in vivo Cre-mediated excision events [18]) and found lacZ expression in the pancreas but not in other organs. Expression was, however, also evident in ∼10% of the beta cells. It is noteworthy that *CEL* expression has not been reported in human pancreatic beta cells [19].

Next, we confirmed murine pancreatic expression of the human *CEL* gene by real-time quantitative PCR. We found normal pancreatic exocrine function, glucose tolerance and pancreatic morphology in the normal chow fed state in both male and female TgCEL mice,(taking into account potential gender differences in the penetrance of a diabetic phenotype [20], [21]), and the pancreatic protein expression pattern was similar in TgCEL and control mice. To further study the possibility of a subtle diabetic phenotype, we introduced a high-fat diet with a 60% fat by calorie (HFD) for twelve weeks which resulted in a substantial body weight gain, increased fasting blood glucose values and reduced glucose tolerance in both groups of mice indicating that the diet did indeed challenge the glucose homeostasis in the animals. However, there were no differences between the groups among any of the measured variables of glucose homeostasis, contrasting the findings in human *CEL* carriers who develop reduced insulin secretion in the second decade as measured by intravenous glucose tolerance tests and subsequently develop diabetes [1]. Analyses at the mRNA level showed no differences in the expression levels of insulin, the glucose transporter Glut2, or any of the transcription factors tested in isolated islets. Further, the size and number of islets were similar in TgCEL and control mice. As the diabetes onset in *CEL*-MODY is relatively late with an onset around 35 years of age [1], we followed the TgCEL mice after cessation of the high fat challenge until an age of 22 months, but did not detect differences in glucose tolerance between groups. Whether these differences in phenotype are due to differential responses between humans and mice requires further detailed investigation, as the genetic background of a mouse model may influence the emergence of a phenotype [16], [21], [22], [23]. As such we cannot rule out the possibility that the absent mutation-specific diabetic phenotype might be related to the C57BL/6-FVB genetic background of these mice.

Human *CEL* mutation carriers develop evidence of pancreatic exocrine disease preceding diabetes and pancreatic lipomatosis [1], [24]. Since pancreatic lipomatosis is a clinical hallmark also in other monogenic pancreatic conditions such as Johanson-Blizzard syndrome and cystic fibrosis [25] and pancreatic inflammation plays a role in the pathogenesis [9], [26], we exposed the TgCEL mice to cerulein to elicit pancreatic inflammation. The decision to choose cerulein was based on the observation that it has been previously successful in triggering pancreatitis in murine models [12], including a murine model for Johanson-Blizzard syndrome [9]. However, we did not detect mutation-specific differences in the serum levels of serum amylase as an indicator of acute pancreatitis after short-term cerulein exposure or in the extent of pancreatic tissue inflammation after short-term or long-term cerulein exposure. Indeed, the cerulein-exposed TgCEL mice gained weight normally and remained glucose tolerant. Thus, neither a HFD or cerulein challenge unmasked signs of pancreatic exocrine dysfunction in the TgCEL mice, which had normal weight gain, normal lipids and lipid-soluble vitamins, normal fecal elastase and normal fat absorption contrasting the steatorrea and reduced levels of fecal elastase and lipid-soluble vitamins observed in human *CEL* mutation carriers [1].

Several explanations are possible for the absence of a pancreatic phenotype in the TgCEL mice. First, we applied pronuclear injection to create transgenic mice which can result in a multiple array of transgenes [27], and it has been reported that a high number of transgenes can lead to silencing of gene expression [28]. Thus, the absent pancreatic phenotype could be explained by a high copy number of the transgene. We have confirmed pancreatic expression of the transgene at the mRNA level, but the copy number of the transgene and the protein expression relative to the wild type allele remains to be studied. Since knock-in mice carry the mutation in its appropriate genomic and protein context, a knock-in model would have potentially been a more faithful genetic model of CEL-MODY [29].

Second, it is possible that an embryonically pancreatic exocrine expression of the disease-associated allele earlier than day e14–e15 achieved with elastase-Cre [30] would potentially have elicited a pancreatic phenotype. This could be achieved by crossing floxed CEL mice with Cre mouse lines driven by other promoters such as ptf1-Cre since *Ptf1a* is expressed as early as day e9.5 [31]. Alternatively, ubiquitous organ expression using CMV-Cre would allow the exploration of the possibility that other organ systems are involved in the pathogenesis of CEL-MODY. Third, many late-onset human phenotypes may not be apparent in mice [32], [33]. Fourth, the absent pancreatic phenotype may be caused by species-specific differences. There are species-specific differences in the pancreas-specific regulation of murine and human *CEL* expression [34] and rodent *Cel* genes have a remarkably shorter exon 11 VNTR than human subjects [35]. Since protein misfolding may play a role in the pathogenic process [3] and species-specific differences in the unfolded protein response have been observed, at least in lower eukaryotes [36], similar species-specific differences between mouse and man may explain the negative findings. In addition, human *CEL*, in contrast to the murine *Cel*, is present in plasma [37], [38] probably by expression in human endothelial cells [39] and may cause systemic effects potentially affecting the pancreatic phenotype.

In conclusion, we have created a transgenic mouse line, the TgCEL, with pancreas-specific expression of human *CEL* with the mutation of a family of *CEL* mutation carriers [1]. The lack of a distinct phenotype in this model, even when challenged by a HFD or cerulein exposure, may be secondary to species-specific mechanisms preventing the human phenotype from emerging in TgCEL mice or to properties of the genetic background or the promoter. The latter possibilities warrant studies of TgCEL mice on different genetic backgrounds or with Cre driven by a different promoter.

## Supporting Information

Supporting Information S1 **Primer information for quantitative PCR experiments.** Quantitative proteomics of murine pancreatic tissue using TMT labeling.
(DOCX)

Supporting Information S2 **Protein abundance in TgCEL and control mice.** Heatmap from an unsupervised analysis of protein abundance in TgCEL and control mice based on a quantitative proteomics method using isobaric labeling with tandem mass tags (TMT) and LC-MS/MS analysis on an an LTQ-Orbitrap Elite mass spectrometer. The coding of the mice was as follows: 1: 126 = TgCEL, PBS, 2: 127a = Control, PBS, 3: 127b = TgCEL, Cerulein, 4: 128 = Control, Cerulein, 5: 129a = Control, PBS, 6: 129b = TgCEL, PBS, 7: 130 = TgCEL, Cerulein, 8: 131 = Control, Cerulein.
(TIF)
