# Supplementary material for: Absence of Diabetes and Pancreatic Exocrine Dysfunction in a Transgenic Model of Carboxyl-Ester Lipase-MODY (Maturity-Onset Diabetes of the Young)
Source: PLoS One. 2013 Apr 2;8(4):e60229. doi: 10.1371/journal.pone.0060229 (PMC3615023; doi:10.1371/journal.pone.0060229)
Supplement: Supporting Information S1 — Primer information for quantitative PCR experiments. Quantitative proteomics of murine pancreatic tissue using TMT labeling. (DOCX) [file pone.0060229.s001.docx]

**Supporting Information Methods and Legends S1**

**Raeder et al**

**Primer information for quantitative PCR experiments**

Exocrine enzymes:

Human CEL Hs00426932_m1 Taqman (human)

Mouse CEL Mm00486975_m1 Taqman (mouse)

Transcription factors:

PDX-1 F 5' cct ttc cca tgg atg aag tc 3'

PDX-1 R 5' ttc aac atg aca gcc agc tc 3'

Maf-a F 5' aaa agc ggt gct gga gga t 3'

Maf-a R 5' tcc tcc ggc gtc agg tt 3'

Neuro-D1 F 5' cca tgg tgg gtt gtc ata tat tc 3'

Neuro-D1 R 5' cag cat cac atc tca aac agc 3'

HNF-1a F 5' ctt gtt tgg ggc agg agt ag 3'

HNF-1a R 5' ggt tca cag gct cct ttg tc 3'

HNF-4a F 5' cag atg atc gag cag atc ca 3'

HNF-4a R 5' cgt tgg ttc cca tat gtt cc 3'

Glucose homeostasis:

Mouse Insulin-1 F 5' cct gtt ggt gca ctt cct a 3'

Mouse Insulin-1 R 5' tct gaa ggt ccc cgg ggc t 3'

Glucagon F 5' tgg tgc tca tct cgt cag ag 3'

Glucagon R 5' tga att tga gag gca tgc tg 3'

Gck F3 5' aac caa ctt cag ggt gat gc 3'

Gck R3 5' agg gaa gga gaa ggt gaa gc 3'

GLUT-2 F 5' ggc aca tcc tac ttg gcc ta 3'

GLUT-2 R 5' aga cct tct gct cag tcg at 3'

**Quantitative proteomics of murine pancreatic tissue using TMT labeling**

Murine pancreatic tissue was lysed using 10 mM K2HPO4 pH 7.5, 1 mM EDTA, 10 mM MgCl2, 50 mM β-glycerophosphate, 5 mM EGTA, 0.5% Nonidet P-40, 0.1% Brij 35, 0.1% deoxycholic acid, 1mM sodium orthovanadate, 1 mM phenylmethyl-sulfonyl fluoride, 5 g/ml leupeptin and 5 g/ml pepstatin A. The protein concentration was determined using Bradford protein assay (Pierce). The lysate (100 μg) from 4 TgCEL mice and 4 control mice was purified using methanol/chloroform precipitation. The purified protein pellets were each resuspended in 100 μL of 50 mM HEPES, 4M Urea, pH 8.5. The cysteine residues were reduced and alkylated with DTT (5 mM) and iodoacetamide (15 mM) respectively prior to digestion with Lys-C (Wako, Japan) in a 1:100 LysC:protein ratio. Following digestion overnight, the digest was acidified with formic acid prior to desalting with C18 solid-phase extraction (Sep-Pak, Waters, Milford, MA). The desalted peptides were then resuspended in 100 μL of 50 mM HEPES, pH 8.5 and each sample was labeled with one of the 8-plex tandem mass tag (TMT) reagents (Thermo Scientific, Rockford, IL) for one hour at room temperature. The TMT labeled peptides were purified, fractionated by high pH reversed-phase chromatography and 12 fractions were analysed by LC-MS/MS on an LTQ-Orbitrap Elite mass spectrometer.

**Legend Supplementary Figure S2**

**Protein abundance in TgCEL and control mice.** Heatmap from an unsupervised analysis of protein abundance in TgCEL and control mice based on a quantitative proteomics method using isobaric labeling with tandem mass tags (TMT) and LC-MS/MS analysis on an an LTQ-Orbitrap Elite mass spectrometer. The coding of the mice was as follows:

1: 126 = TgCEL, PBS

2: 127a = Control, PBS

3: 127b = TgCEL, Cerulein

4: 128 = Control, Cerulein

5: 129a = Control, PBS

6: 129b = TgCEL, PBS

7: 130 = TgCEL, Cerulein

8: 131 = Control, Cerulein
